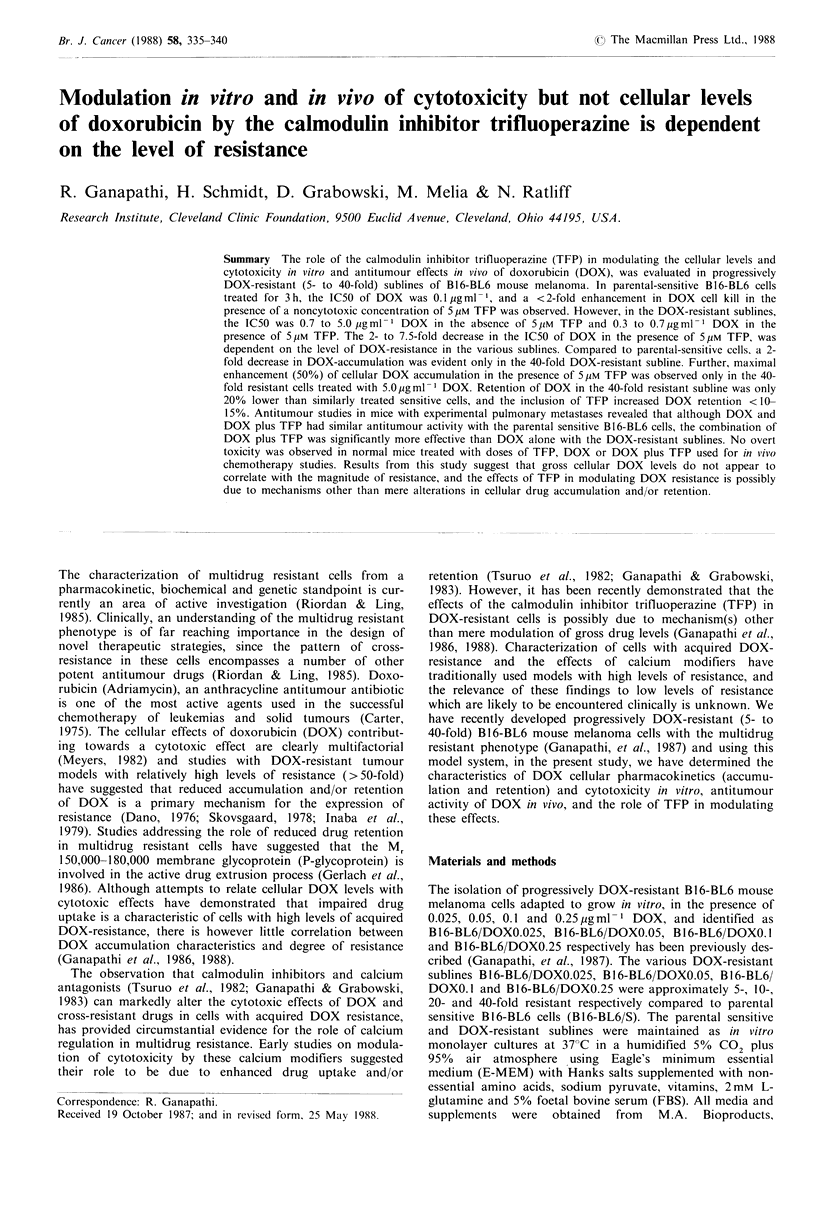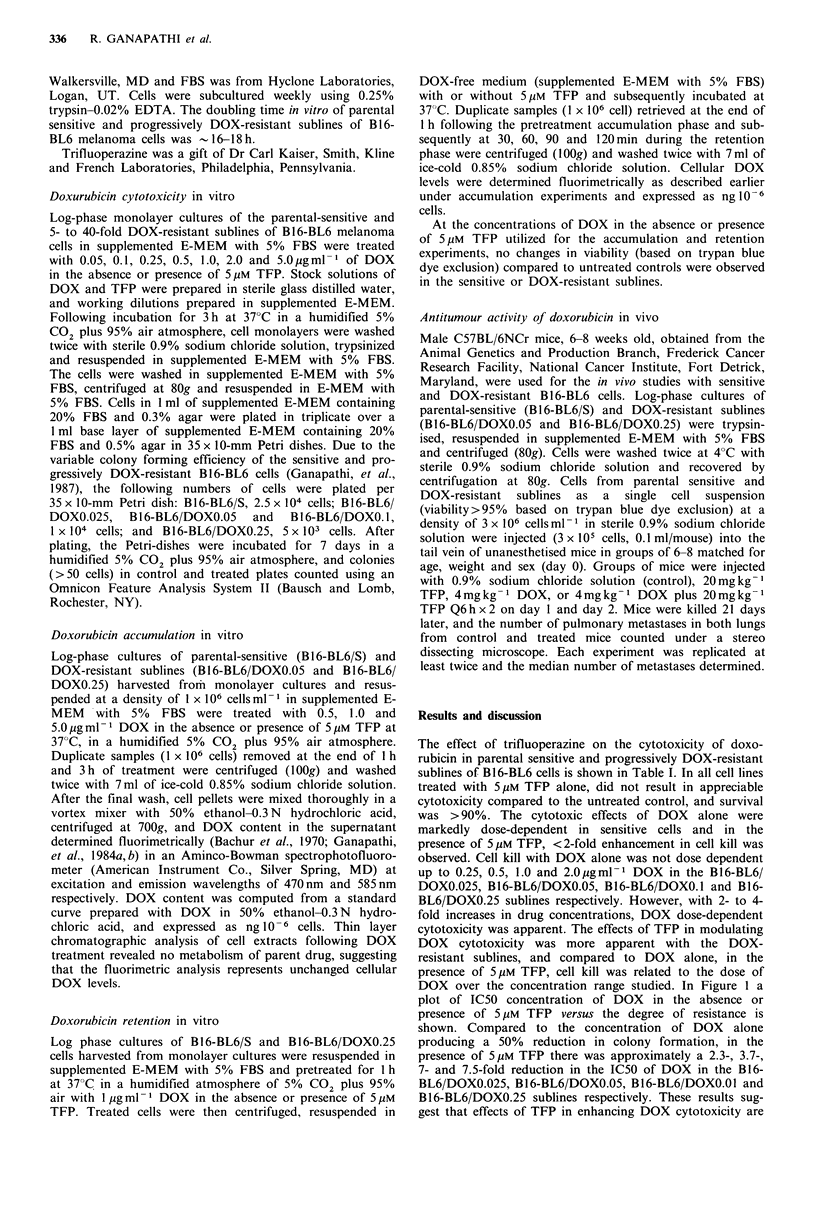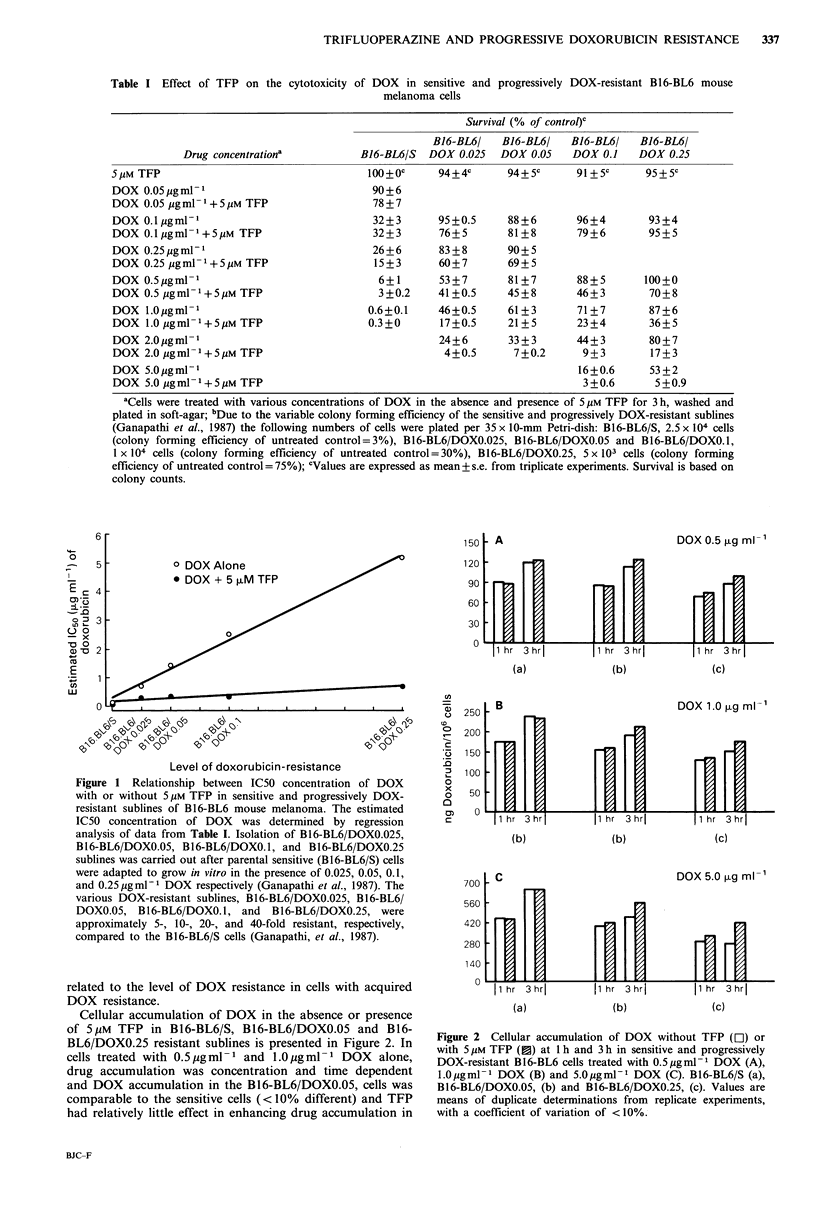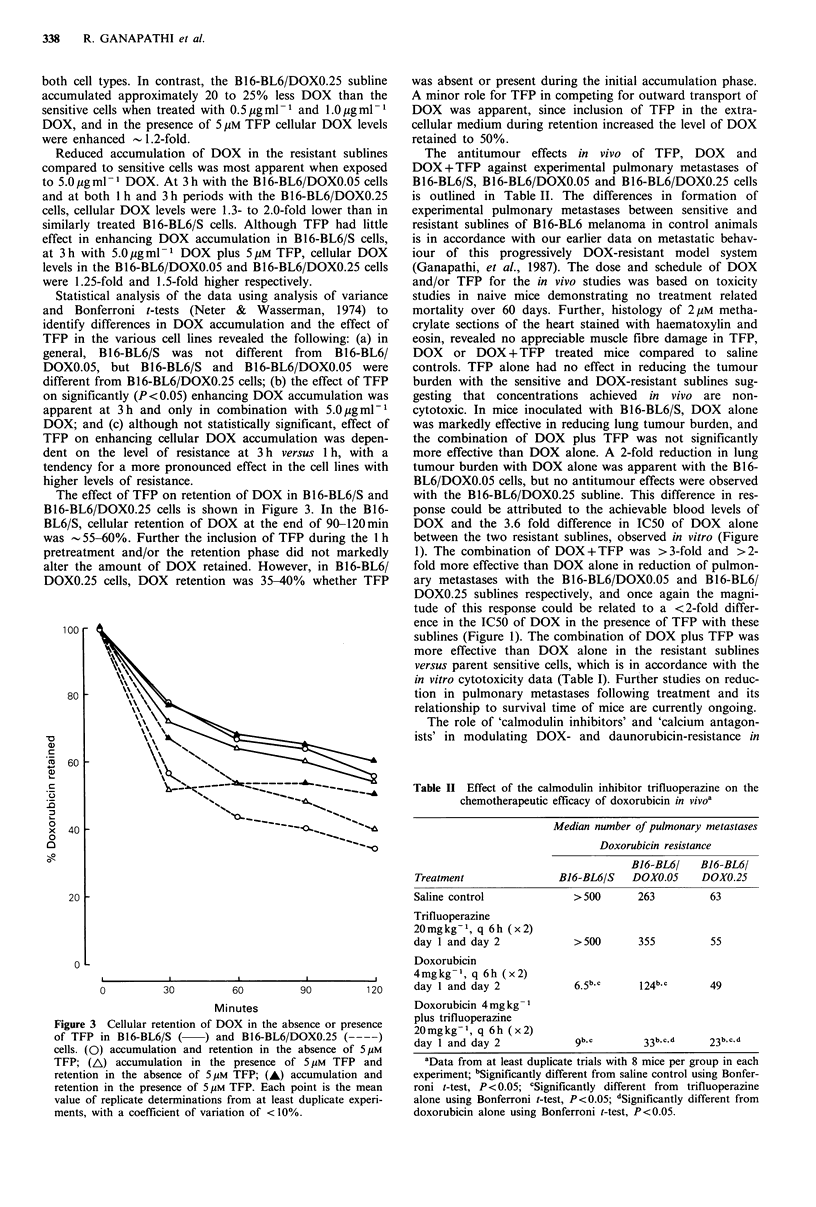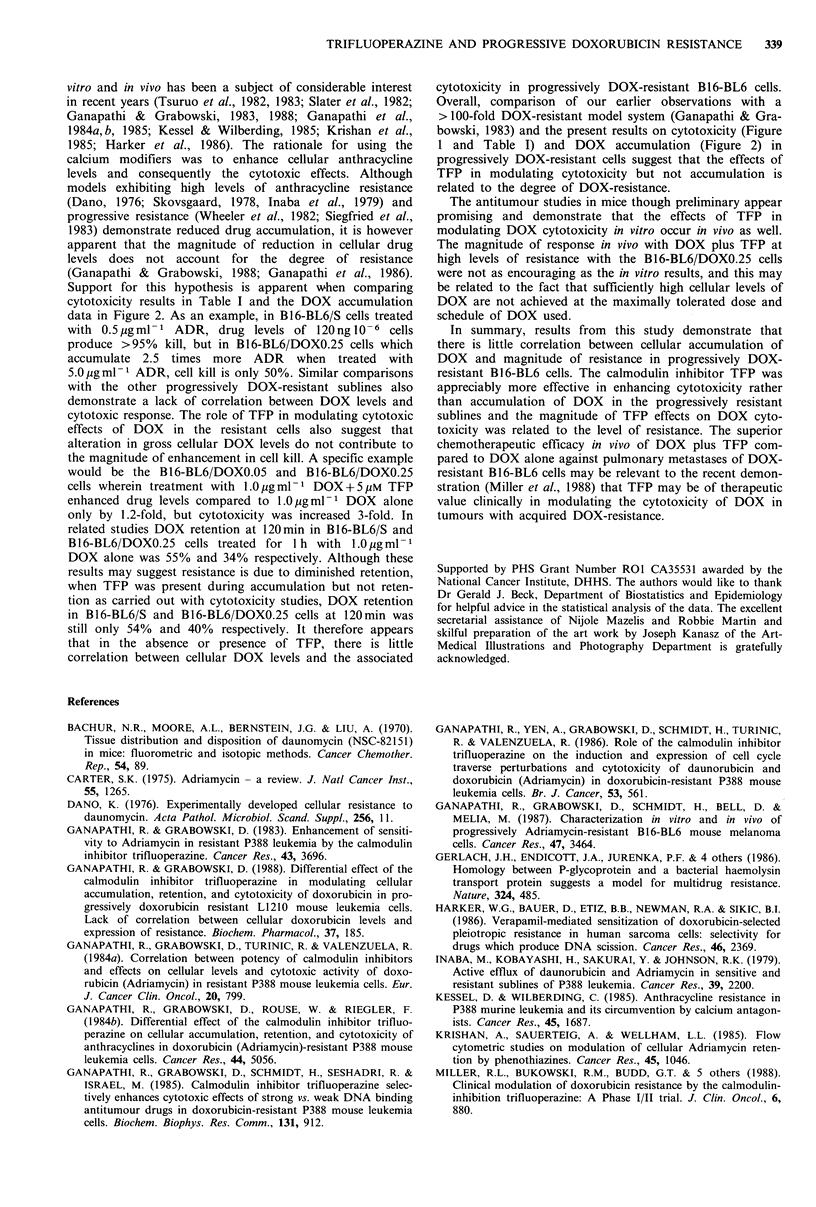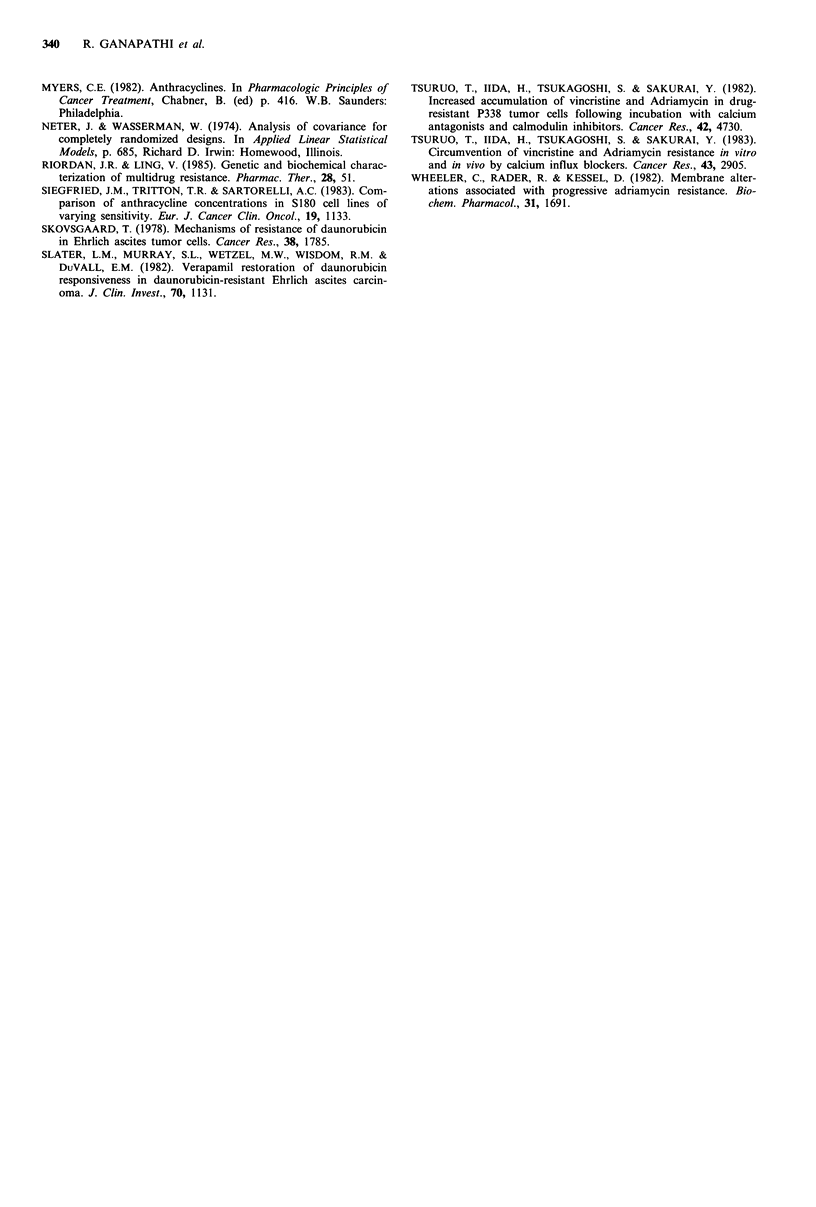# Modulation in vitro and in vivo of cytotoxicity but not cellular levels of doxorubicin by the calmodulin inhibitor trifluoperazine is dependent on the level of resistance.

**DOI:** 10.1038/bjc.1988.214

**Published:** 1988-09

**Authors:** R. Ganapathi, H. Schmidt, D. Grabowski, M. Melia, N. Ratliff

**Affiliations:** Research Institute, Cleveland Clinic Foundation, Ohio 44195.

## Abstract

The role of the calmodulin inhibitor trifluoperazine (TFP) in modulating the cellular levels and cytotoxicity in vitro and antitumour effects in vivo of doxorubicin (DOX), was evaluated in progressively DOX-resistant (5- to 40-fold) sublines of B16-BL6 mouse melanoma. In parental-sensitive B16-BL6 cells treated for 3 h, the IC50 of DOX was 0.1 microgram ml-1, and a less than 2-fold enhancement in DOX cell kill in the presence of a noncytotoxic concentration of 5 microM TFP was observed. However, in the DOX-resistant sublines, the IC50 was 0.7 to 5.0 micrograms ml-1 DOX in the absence of 5 microM TFP and 0.3 to 0.7 microgram ml-1 DOX in the presence of 5 microM TFP. The 2- to 7.5-fold decrease in the IC50 of DOX in the presence of 5 microM TFP, was dependent on the level of DOX-resistance in the various sublines. Compared to parental-sensitive cells, a 2-fold decrease in DOX-accumulation was evident only in the 40-fold DOX-resistant subline. Further, maximal enhancement (50%) of cellular DOX accumulation in the presence of 5 microM TFP was observed only in the 40-fold resistant cells treated with 5.0 micrograms ml-1 DOX. Retention of DOX in the 40-fold resistant subline was only 20% lower than similarly treated sensitive cells, and the inclusion of TFP increased DOX retention less than 10-15%. Antitumour studies in mice with experimental pulmonary metastases revealed that although DOX and DOX plus TFP had similar antitumour activity with the parental sensitive B16-BL6 cells, the combination of DOX plus TFP was significantly more effective than DOX alone with the DOX-resistant sublines. No overt toxicity was observed in normal mice treated with doses of TFP, DOX or DOX plus TFP used for in vivo chemotherapy studies. Results from this study suggest that gross cellular DOX levels do not appear to correlate with the magnitude of resistance, and the effects of TFP in modulating DOX resistance is possibly due to mechanisms other than mere alterations in cellular drug accumulation and/or retention.


*Br. J. Cancer* (1988) **58**, 335–340 © The Macmillan Press Ltd., 1988

### Modulation *in vitro* and *in vivo* of cytotoxicity but not cellular levels of doxorubicin by the calmodulin inhibitor trifluoperazine is dependent on the level of resistance

R. Ganapathi, H. Schmidt, D. Grabowski, M. Melia & N. Ratliff

*Research Institute, Cleveland Clinic Foundation, 9500 Euclid Avenue, Cleveland, Ohio 44195, USA.*

**Summary** The role of the calmodulin inhibitor trifluoperazine (TFP) in modulating the cellular levels and cytotoxicity *in vitro* and antitumour effects *in vivo* of doxorubicin (DOX), was evaluated in progressively DOX-resistant (5- to 40-fold) sublines of B16-BL6 mouse melanoma. In parental-sensitive B16-BL6 cells treated for 3 h, the IC50 of DOX was $0.1\,\mu g\,ml^{-1}$, and a <2-fold enhancement in DOX cell kill in the presence of a noncytotoxic concentration of $5\,\mu M$ TFP was observed. However, in the DOX-resistant sublines, the IC50 was 0.7 to $5.0\,\mu g\,ml^{-1}$ DOX in the absence of $5\,\mu M$ TFP and 0.3 to $0.7\,\mu g\,ml^{-1}$ DOX in the presence of $5\,\mu M$ TFP. The 2- to 7.5-fold decrease in the IC50 of DOX in the presence of $5\,\mu M$ TFP, was dependent on the level of DOX-resistance in the various sublines. Compared to parental-sensitive cells, a 2-fold decrease in DOX-accumulation was evident only in the 40-fold DOX-resistant subline. Further, maximal enhancement (50%) of cellular DOX accumulation in the presence of $5\,\mu M$ TFP was observed only in the 40-fold resistant cells treated with $5.0\,\mu g\,ml^{-1}$ DOX. Retention of DOX in the 40-fold resistant subline was only 20% lower than similarly treated sensitive cells, and the inclusion of TFP increased DOX retention <10–15%. Antitumour studies in mice with experimental pulmonary metastases revealed that although DOX and DOX plus TFP had similar antitumour activity with the parental sensitive B16-BL6 cells, the combination of DOX plus TFP was significantly more effective than DOX alone with the DOX-resistant sublines. No overt toxicity was observed in normal mice treated with doses of TFP, DOX or DOX plus TFP used for *in vivo* chemotherapy studies. Results from this study suggest that gross cellular DOX levels do not appear to correlate with the magnitude of resistance, and the effects of TFP in modulating DOX resistance is possibly due to mechanisms other than mere alterations in cellular drug accumulation and/or retention.

The characterization of multidrug resistant cells from a pharmacokinetic, biochemical and genetic standpoint is currently an area of active investigation (Riordan & Ling, 1985). Clinically, an understanding of the multidrug resistant phenotype is of far reaching importance in the design of novel therapeutic strategies, since the pattern of cross-resistance in these cells encompasses a number of other potent antitumour drugs (Riordan & Ling, 1985). Doxorubicin (Adriamycin), an anthracycline antitumour antibiotic is one of the most active agents used in the successful chemotherapy of leukemias and solid tumours (Carter, 1975). The cellular effects of doxorubicin (DOX) contributing towards a cytotoxic effect are clearly multifactorial (Meyers, 1982) and studies with DOX-resistant tumour models with relatively high levels of resistance (>50-fold) have suggested that reduced accumulation and/or retention of DOX is a primary mechanism for the expression of resistance (Dano, 1976; Skovsgaard, 1978; Inaba *et al.*, 1979). Studies addressing the role of reduced drug retention in multidrug resistant cells have suggested that the $M_r$ 150,000–180,000 membrane glycoprotein (P-glycoprotein) is involved in the active drug extrusion process (Gerlach *et al.*, 1986). Although attempts to relate cellular DOX levels with cytotoxic effects have demonstrated that impaired drug uptake is a characteristic of cells with high levels of acquired DOX-resistance, there is however little correlation between DOX accumulation characteristics and degree of resistance (Ganapathi *et al.*, 1986, 1988).

The observation that calmodulin inhibitors and calcium antagonists (Tsuruo *et al.*, 1982; Ganapathi & Grabowski, 1983) can markedly alter the cytotoxic effects of DOX and cross-resistant drugs in cells with acquired DOX resistance, has provided circumstantial evidence for the role of calcium regulation in multidrug resistance. Early studies on modulation of cytotoxicity by these calcium modifiers suggested their role to be due to enhanced drug uptake and/or retention (Tsuruo *et al.*, 1982; Ganapathi & Grabowski, 1983). However, it has been recently demonstrated that the effects of the calmodulin inhibitor trifluoperazine (TFP) in DOX-resistant cells is possibly due to mechanism(s) other than mere modulation of gross drug levels (Ganapathi *et al.*, 1986, 1988). Characterization of cells with acquired DOX-resistance and the effects of calcium modifiers have traditionally used models with high levels of resistance, and the relevance of these findings to low levels of resistance which are likely to be encountered clinically is unknown. We have recently developed progressively DOX-resistant (5- to 40-fold) B16-BL6 mouse melanoma cells with the multidrug resistant phenotype (Ganapathi, *et al.*, 1987) and using this model system, in the present study, we have determined the characteristics of DOX cellular pharmacokinetics (accumulation and retention) and cytotoxicity *in vitro*, antitumour activity of DOX *in vivo*, and the role of TFP in modulating these effects.

Correspondence: R. Ganapathi.
Received 19 October 1987; and in revised form, 25 May 1988.

### Materials and methods

The isolation of progressively DOX-resistant B16-BL6 mouse melanoma cells adapted to grow *in vitro*, in the presence of 0.025, 0.05, 0.1 and $0.25\,\mu g\,ml^{-1}$ DOX, and identified as B16-BL6/DOX0.025, B16-BL6/DOX0.05, B16-BL6/DOX0.1 and B16-BL6/DOX0.25 respectively has been previously described (Ganapathi, *et al.*, 1987). The various DOX-resistant sublines B16-BL6/DOX0.025, B16-BL6/DOX0.05, B16-BL6/DOX0.1 and B16-BL6/DOX0.25 were approximately 5-, 10-, 20- and 40-fold resistant respectively compared to parental sensitive B16-BL6 cells (B16-BL6/S). The parental sensitive and DOX-resistant sublines were maintained as *in vitro* monolayer cultures at 37°C in a humidified 5% $CO_2$ plus 95% air atmosphere using Eagle's minimum essential medium (E-MEM) with Hanks salts supplemented with non-essential amino acids, sodium pyruvate, vitamins, 2 mM L-glutamine and 5% foetal bovine serum (FBS). All media and supplements were obtained from M.A. Bioproducts,

Walkersville, MD and FBS was from Hyclone Laboratories, Logan, UT. Cells were subcultured weekly using 0.25% trypsin–0.02% EDTA. The doubling time *in vitro* of parental sensitive and progressively DOX-resistant sublines of B16-BL6 melanoma cells was ~16–18 h.

Trifluoperazine was a gift of Dr Carl Kaiser, Smith, Kline and French Laboratories, Philadelphia, Pennsylvania.

*Doxurubicin cytotoxicity* in vitro

Log-phase monolayer cultures of the parental-sensitive and 5- to 40-fold DOX-resistant sublines of B16-BL6 melanoma cells in supplemented E-MEM with 5% FBS were treated with 0.05, 0.1, 0.25, 0.5, 1.0, 2.0 and 5.0 $\mu g ml^{-1}$ of DOX in the absence or presence of 5 $\mu M$ TFP. Stock solutions of DOX and TFP were prepared in sterile glass distilled water, and working dilutions prepared in supplemented E-MEM. Following incubation for 3 h at 37°C in a humidified 5% $CO_2$ plus 95% air atmosphere, cell monolayers were washed twice with sterile 0.9% sodium chloride solution, trypsinized and resuspended in supplemented E-MEM with 5% FBS. The cells were washed in supplemented E-MEM with 5% FBS, centrifuged at 80*g* and resuspended in E-MEM with 5% FBS. Cells in 1 ml of supplemented E-MEM containing 20% FBS and 0.3% agar were plated in triplicate over a 1 ml base layer of supplemented E-MEM containing 20% FBS and 0.5% agar in 35 × 10-mm Petri dishes. Due to the variable colony forming efficiency of the sensitive and progressively DOX-resistant B16-BL6 cells (Ganapathi, *et al.*, 1987), the following numbers of cells were plated per 35 × 10-mm Petri dish: B16-BL6/S, $2.5 \times 10^4$ cells; B16-BL6/DOX0.025, B16-BL6/DOX0.05 and B16-BL6/DOX0.1, $1 \times 10^4$ cells; and B16-BL6/DOX0.25, $5 \times 10^3$ cells. After plating, the Petri-dishes were incubated for 7 days in a humidified 5% $CO_2$ plus 95% air atmosphere, and colonies (> 50 cells) in control and treated plates counted using an Omnicon Feature Analysis System II (Bausch and Lomb, Rochester, NY).

*Doxorubicin accumulation* in vitro

Log-phase cultures of parental-sensitive (B16-BL6/S) and DOX-resistant sublines (B16-BL6/DOX0.05 and B16-BL6/DOX0.25) harvested from monolayer cultures and resuspended at a density of $1 \times 10^6$ cells $ml^{-1}$ in supplemented E-MEM with 5% FBS were treated with 0.5, 1.0 and 5.0 $\mu g ml^{-1}$ DOX in the absence or presence of 5 $\mu M$ TFP at 37°C, in a humidified 5% $CO_2$ plus 95% air atmosphere. Duplicate samples ($1 \times 10^6$ cells) removed at the end of 1 h and 3 h of treatment were centrifuged (100*g*) and washed twice with 7 ml of ice-cold 0.85% sodium chloride solution. After the final wash, cell pellets were mixed thoroughly in a vortex mixer with 50% ethanol–0.3 N hydrochloric acid, centrifuged at 700*g*, and DOX content in the supernatant determined fluorimetrically (Bachur *et al.*, 1970; Ganapathi, *et al.*, 1984*a*,*b*) in an Aminco-Bowman spectrophotofluorometer (American Instrument Co., Silver Spring, MD) at excitation and emission wavelengths of 470 nm and 585 nm respectively. DOX content was computed from a standard curve prepared with DOX in 50% ethanol–0.3 N hydrochloric acid, and expressed as ng $10^{-6}$ cells. Thin layer chromatographic analysis of cell extracts following DOX treatment revealed no metabolism of parent drug, suggesting that the fluorimetric analysis represents unchanged cellular DOX levels.

*Doxorubicin retention* in vitro

Log phase cultures of B16-BL6/S and B16-BL6/DOX0.25 cells harvested from monolayer cultures were resuspended in supplemented E-MEM with 5% FBS and pretreated for 1 h at 37°C in a humidified atmosphere of 5% $CO_2$ plus 95% air with 1 $\mu g ml^{-1}$ DOX in the absence or presence of 5 $\mu M$ TFP. Treated cells were then centrifuged, resuspended in DOX-free medium (supplemented E-MEM with 5% FBS) with or without 5 $\mu M$ TFP and subsequently incubated at 37°C. Duplicate samples ($1 \times 10^6$ cell) retrieved at the end of 1 h following the pretreatment accumulation phase and subsequently at 30, 60, 90 and 120 min during the retention phase were centrifuged (100*g*) and washed twice with 7 ml of ice-cold 0.85% sodium chloride solution. Cellular DOX levels were determined fluorimetrically as described earlier under accumulation experiments and expressed as ng $10^{-6}$ cells.

At the concentrations of DOX in the absence or presence of 5 $\mu M$ TFP utilized for the accumulation and retention experiments, no changes in viability (based on trypan blue dye exclusion) compared to untreated controls were observed in the sensitive or DOX-resistant sublines.

*Antitumour activity of doxorubicin* in vivo

Male C57BL/6NCr mice, 6–8 weeks old, obtained from the Animal Genetics and Production Branch, Frederick Cancer Research Facility, National Cancer Institute, Fort Detrick, Maryland, were used for the *in vivo* studies with sensitive and DOX-resistant B16-BL6 cells. Log-phase cultures of parental-sensitive (B16-BL6/S) and DOX-resistant sublines (B16-BL6/DOX0.05 and B16-BL6/DOX0.25) were trypsinised, resuspended in supplemented E-MEM with 5% FBS and centrifuged (80*g*). Cells were washed twice at 4°C with sterile 0.9% sodium chloride solution and recovered by centrifugation at 80*g*. Cells from parental sensitive and DOX-resistant sublines as a single cell suspension (viability > 95% based on trypan blue dye exclusion) at a density of $3 \times 10^6$ cells $ml^{-1}$ in sterile 0.9% sodium chloride solution were injected ($3 \times 10^5$ cells, 0.1 ml/mouse) into the tail vein of unanesthetised mice in groups of 6–8 matched for age, weight and sex (day 0). Groups of mice were injected with 0.9% sodium chloride solution (control), 20 $mg kg^{-1}$ TFP, 4 $mg kg^{-1}$ DOX, or 4 $mg kg^{-1}$ DOX plus 20 $mg kg^{-1}$ TFP Q6 h × 2 on day 1 and day 2. Mice were killed 21 days later, and the number of pulmonary metastases in both lungs from control and treated mice counted under a stereo dissecting microscope. Each experiment was replicated at least twice and the median number of metastases determined.

### Results and discussion

The effect of trifluoperazine on the cytotoxicity of doxorubicin in parental sensitive and progressively DOX-resistant sublines of B16-BL6 cells is shown in Table I. In all cell lines treated with 5 $\mu M$ TFP alone, did not result in appreciable cytotoxicity compared to the untreated control, and survival was >90%. The cytotoxic effects of DOX alone were markedly dose-dependent in sensitive cells and in the presence of 5 $\mu M$ TFP, <2-fold enhancement in cell kill was observed. Cell kill with DOX alone was not dose dependent up to 0.25, 0.5, 1.0 and 2.0 $\mu g ml^{-1}$ DOX in the B16-BL6/DOX0.025, B16-BL6/DOX0.05, B16-BL6/DOX0.1 and B16-BL6/DOX0.25 sublines respectively. However, with 2- to 4-fold increases in drug concentrations, DOX dose-dependent cytotoxicity was apparent. The effects of TFP in modulating DOX cytotoxicity was more apparent with the DOX-resistant sublines, and compared to DOX alone, in the presence of 5 $\mu M$ TFP, cell kill was related to the dose of DOX over the concentration range studied. In Figure 1 a plot of IC50 concentration of DOX in the absence or presence of 5 $\mu M$ TFP *versus* the degree of resistance is shown. Compared to the concentration of DOX alone producing a 50% reduction in colony formation, in the presence of 5 $\mu M$ TFP there was approximately a 2.3-, 3.7-, 7- and 7.5-fold reduction in the IC50 of DOX in the B16-BL6/DOX0.025, B16-BL6/DOX0.05, B16-BL6/DOX0.01 and B16-BL6/DOX0.25 sublines respectively. These results suggest that effects of TFP in enhancing DOX cytotoxicity are

**Table I** Effect of TFP on the cytotoxicity of DOX in sensitive and progressively DOX-resistant B16-BL6 mouse melanoma cells

| | *Survival (% of control)*[c] | | | | |
|---|---|---|---|---|---|
| *Drug concentration*[a] | *B16-BL6/S* | *B16-BL6/ DOX 0.025* | *B16-BL6/ DOX 0.05* | *B16-BL6/ DOX 0.1* | *B16-BL6/ DOX 0.25* |
| 5 μM TFP | 100±0[c] | 94±4[c] | 94±5[c] | 91±5[c] | 95±5[c] |
| DOX 0.05 μg ml$^{-1}$ | 90±6 | | | | |
| DOX 0.05 μg ml$^{-1}$ + 5 μM TFP | 78±7 | | | | |
| DOX 0.1 μg ml$^{-1}$ | 32±3 | 95±0.5 | 88±6 | 96±4 | 93±4 |
| DOX 0.1 μg ml$^{-1}$ + 5 μM TFP | 32±3 | 76±5 | 81±8 | 79±6 | 95±5 |
| DOX 0.25 μg ml$^{-1}$ | 26±6 | 83±8 | 90±5 | | |
| DOX 0.25 μg ml$^{-1}$ + 5 μM TFP | 15±3 | 60±7 | 69±5 | | |
| DOX 0.5 μg ml$^{-1}$ | 6±1 | 53±7 | 81±7 | 88±5 | 100±0 |
| DOX 0.5 μg ml$^{-1}$ + 5 μM TFP | 3±0.2 | 41±0.5 | 45±8 | 46±3 | 70±8 |
| DOX 1.0 μg ml$^{-1}$ | 0.6±0.1 | 46±0.5 | 61±3 | 71±7 | 87±6 |
| DOX 1.0 μg ml$^{-1}$ + 5 μM TFP | 0.3±0 | 17±0.5 | 21±5 | 23±4 | 36±5 |
| DOX 2.0 μg ml$^{-1}$ | | 24±6 | 33±3 | 44±3 | 80±7 |
| DOX 2.0 μg ml$^{-1}$ + 5 μM TFP | | 4±0.5 | 7±0.2 | 9±3 | 17±3 |
| DOX 5.0 μg ml$^{-1}$ | | | | 16±0.6 | 53±2 |
| DOX 5.0 μg ml$^{-1}$ + 5 μM TFP | | | | 3±0.6 | 5±0.9 |

[a]Cells were treated with various concentrations of DOX in the absence and presence of 5 μM TFP for 3 h, washed and plated in soft-agar; [b]Due to the variable colony forming efficiency of the sensitive and progressively DOX-resistant sublines (Ganapathi *et al.*, 1987) the following numbers of cells were plated per 35 × 10-mm Petri-dish: B16-BL6/S, $2.5 \times 10^4$ cells (colony forming efficiency of untreated control = 3%), B16-BL6/DOX0.025, B16-BL6/DOX0.05 and B16-BL6/DOX0.1, $1 \times 10^4$ cells (colony forming efficiency of untreated control = 30%), B16-BL6/DOX0.25, $5 \times 10^3$ cells (colony forming efficiency of untreated control = 75%); [c]Values are expressed as mean ± s.e. from triplicate experiments. Survival is based on colony counts.

**Figure 1** Relationship between IC50 concentration of DOX with or without 5 μM TFP in sensitive and progressively DOX-resistant sublines of B16-BL6 mouse melanoma. The estimated IC50 concentration of DOX was determined by regression analysis of data from **Table I**. Isolation of B16-BL6/DOX0.025, B16-BL6/DOX0.05, B16-BL6/DOX0.1, and B16-BL6/DOX0.25 sublines was carried out after parental sensitive (B16-BL6/S) cells were adapted to grow *in vitro* in the presence of 0.025, 0.05, 0.1, and 0.25 μg ml$^{-1}$ DOX respectively (Ganapathi *et al.*, 1987). The various DOX-resistant sublines, B16-BL6/DOX0.025, B16-BL6/DOX0.05, B16-BL6/DOX0.1, and B16-BL6/DOX0.25, were approximately 5-, 10-, 20-, and 40-fold resistant, respectively, compared to the B16-BL6/S cells (Ganapathi, *et al.*, 1987).

**Figure 2** Cellular accumulation of DOX without TFP (□) or with 5 μM TFP (▨) at 1 h and 3 h in sensitive and progressively DOX-resistant B16-BL6 cells treated with 0.5 μg ml$^{-1}$ DOX (A), 1.0 μg ml$^{-1}$ DOX (B) and 5.0 μg ml$^{-1}$ DOX (C). B16-BL6/S (a), B16-BL6/DOX0.05, (b) and B16-BL6/DOX0.25, (c). Values are means of duplicate determinations from replicate experiments, with a coefficient of variation of < 10%.

related to the level of DOX resistance in cells with acquired DOX resistance.

Cellular accumulation of DOX in the absence or presence of 5 μM TFP in B16-BL6/S, B16-BL6/DOX0.05 and B16-BL6/DOX0.25 resistant sublines is presented in Figure 2. In cells treated with 0.5 μg ml$^{-1}$ and 1.0 μg ml$^{-1}$ DOX alone, drug accumulation was concentration and time dependent and DOX accumulation in the B16-BL6/DOX0.05, cells was comparable to the sensitive cells (< 10% different) and TFP had relatively little effect in enhancing drug accumulation in both cell types. In contrast, the B16-BL6/DOX0.25 subline accumulated approximately 20 to 25% less DOX than the sensitive cells when treated with $0.5\,\mu g\,ml^{-1}$ and $1.0\,\mu g\,ml^{-1}$ DOX, and in the presence of $5\,\mu M$ TFP cellular DOX levels were enhanced ~1.2-fold.

Reduced accumulation of DOX in the resistant sublines compared to sensitive cells was most apparent when exposed to $5.0\,\mu g\,ml^{-1}$ DOX. At 3 h with the B16-BL6/DOX0.05 cells and at both 1 h and 3 h periods with the B16-BL6/DOX0.25 cells, cellular DOX levels were 1.3- to 2.0-fold lower than in similarly treated B16-BL6/S cells. Although TFP had little effect in enhancing DOX accumulation in B16-BL6/S cells, at 3 h with $5.0\,\mu g\,ml^{-1}$ DOX plus $5\,\mu M$ TFP, cellular DOX levels in the B16-BL6/DOX0.05 and B16-BL6/DOX0.25 cells were 1.25-fold and 1.5-fold higher respectively.

Statistical analysis of the data using analysis of variance and Bonferroni *t*-tests (Neter & Wasserman, 1974) to identify differences in DOX accumulation and the effect of TFP in the various cell lines revealed the following: (a) in general, B16-BL6/S was not different from B16-BL6/DOX0.05, but B16-BL6/S and B16-BL6/DOX0.05 were different from B16-BL6/DOX0.25 cells; (b) the effect of TFP on significantly ($P<0.05$) enhancing DOX accumulation was apparent at 3 h and only in combination with $5.0\,\mu g\,ml^{-1}$ DOX; and (c) although not statistically significant, effect of TFP on enhancing cellular DOX accumulation was dependent on the level of resistance at 3 h *versus* 1 h, with a tendency for a more pronounced effect in the cell lines with higher levels of resistance.

The effect of TFP on retention of DOX in B16-BL6/S and B16-BL6/DOX0.25 cells is shown in Figure 3. In the B16-BL6/S, cellular retention of DOX at the end of 90–120 min was ~55–60%. Further the inclusion of TFP during the 1 h pretreatment and/or the retention phase did not markedly alter the amount of DOX retained. However, in B16-BL6/DOX0.25 cells, DOX retention was 35–40% whether TFP was absent or present during the initial accumulation phase. A minor role for TFP in competing for outward transport of DOX was apparent, since inclusion of TFP in the extracellular medium during retention increased the level of DOX retained to 50%.

**Figure 3** Cellular retention of DOX in the absence or presence of TFP in B16-BL6/S (——) and B16-BL6/DOX0.25 (----) cells. (○) accumulation and retention in the absence of $5\,\mu M$ TFP; (△) accumulation in the presence of $5\,\mu M$ TFP and retention in the absence of $5\,\mu M$ TFP; (▲) accumulation and retention in the presence of $5\,\mu M$ TFP. Each point is the mean value of replicate determinations from at least duplicate experiments, with a coefficient of variation of <10%.

The antitumour effects *in vivo* of TFP, DOX and DOX+TFP against experimental pulmonary metastases of B16-BL6/S, B16-BL6/DOX0.05 and B16-BL6/DOX0.25 cells is outlined in Table II. The differences in formation of experimental pulmonary metastases between sensitive and resistant sublines of B16-BL6 melanoma in control animals is in accordance with our earlier data on metastatic behaviour of this progressively DOX-resistant model system (Ganapathi, *et al.*, 1987). The dose and schedule of DOX and/or TFP for the *in vivo* studies was based on toxicity studies in naive mice demonstrating no treatment related mortality over 60 days. Further, histology of $2\,\mu M$ methacrylate sections of the heart stained with haematoxylin and eosin, revealed no appreciable muscle fibre damage in TFP, DOX or DOX+TFP treated mice compared to saline controls. TFP alone had no effect in reducing the tumour burden with the sensitive and DOX-resistant sublines suggesting that concentrations achieved *in vivo* are non-cytotoxic. In mice inoculated with B16-BL6/S, DOX alone was markedly effective in reducing lung tumour burden, and the combination of DOX plus TFP was not significantly more effective than DOX alone. A 2-fold reduction in lung tumour burden with DOX alone was apparent with the B16-BL6/DOX0.05 cells, but no antitumour effects were observed with the B16-BL6/DOX0.25 subline. This difference in response could be attributed to the achievable blood levels of DOX and the 3.6 fold difference in IC50 of DOX alone between the two resistant sublines, observed *in vitro* (Figure 1). The combination of DOX+TFP was >3-fold and >2-fold more effective than DOX alone in reduction of pulmonary metastases with the B16-BL6/DOX0.05 and B16-BL6/DOX0.25 sublines respectively, and once again the magnitude of this response could be related to a <2-fold difference in the IC50 of DOX in the presence of TFP with these sublines (Figure 1). The combination of DOX plus TFP was more effective than DOX alone in the resistant sublines *versus* parent sensitive cells, which is in accordance with the *in vitro* cytotoxicity data (Table I). Further studies on reduction in pulmonary metastases following treatment and its relationship to survival time of mice are currently ongoing.

The role of 'calmodulin inhibitors' and 'calcium antagonists' in modulating DOX- and daunorubicin-resistance *in*

**Table II** Effect of the calmodulin inhibitor trifluoperazine on the chemotherapeutic efficacy of doxorubicin *in vivo*[a]

| | *Median number of pulmonary metastases* | | |
|---|---|---|---|
| | *Doxorubicin resistance* | | |
| *Treatment* | *B16-BL6/S* | *B16-BL6/ DOX0.05* | *B16-BL6/ DOX0.25* |
| Saline control | >500 | 263 | 63 |
| Trifluoperazine $20\,mg\,kg^{-1}$, q 6h (×2) day 1 and day 2 | >500 | 355 | 55 |
| Doxorubicin $4\,mg\,kg^{-1}$, q 6h (×2) day 1 and day 2 | 6.5[b,c] | 124[b,c] | 49 |
| Doxorubicin $4\,mg\,kg^{-1}$ plus trifluoperazine $20\,mg\,kg^{-1}$, q 6h (×2) day 1 and day 2 | 9[b,c] | 33[b,c,d] | 23[b,c,d] |

[a]Data from at least duplicate trials with 8 mice per group in each experiment; [b]Significantly different from saline control using Bonferroni *t*-test, $P<0.05$; [c]Significantly different from trifluoperazine alone using Bonferroni *t*-test, $P<0.05$; [d]Significantly different from doxorubicin alone using Bonferroni *t*-test, $P<0.05$.

*vitro* and *in vivo* has been a subject of considerable interest in recent years (Tsuruo *et al.*, 1982, 1983; Slater *et al.*, 1982; Ganapathi & Grabowski, 1983, 1988; Ganapathi *et al.*, 1984*a,b*, 1985; Kessel & Wilberding, 1985; Krishan *et al.*, 1985; Harker *et al.*, 1986). The rationale for using the calcium modifiers was to enhance cellular anthracycline levels and consequently the cytotoxic effects. Although models exhibiting high levels of anthracycline resistance (Dano, 1976; Skovsgaard, 1978, Inaba *et al.*, 1979) and progressive resistance (Wheeler *et al.*, 1982; Siegfried *et al.*, 1983) demonstrate reduced drug accumulation, it is however apparent that the magnitude of reduction in cellular drug levels does not account for the degree of resistance (Ganapathi & Grabowski, 1988; Ganapathi *et al.*, 1986). Support for this hypothesis is apparent when comparing cytotoxicity results in Table I and the DOX accumulation data in Figure 2. As an example, in B16-BL6/S cells treated with $0.5\,\mu g\,ml^{-1}$ ADR, drug levels of $120\,ng\,10^{-6}$ cells produce >95% kill, but in B16-BL6/DOX0.25 cells which accumulate 2.5 times more ADR when treated with $5.0\,\mu g\,ml^{-1}$ ADR, cell kill is only 50%. Similar comparisons with the other progressively DOX-resistant sublines also demonstrate a lack of correlation between DOX levels and cytotoxic response. The role of TFP in modulating cytotoxic effects of DOX in the resistant cells also suggest that alteration in gross cellular DOX levels do not contribute to the magnitude of enhancement in cell kill. A specific example would be the B16-BL6/DOX0.05 and B16-BL6/DOX0.25 cells wherein treatment with $1.0\,\mu g\,ml^{-1}$ DOX + $5\,\mu M$ TFP enhanced drug levels compared to $1.0\,\mu g\,ml^{-1}$ DOX alone only by 1.2-fold, but cytotoxicity was increased 3-fold. In related studies DOX retention at 120 min in B16-BL6/S and B16-BL6/DOX0.25 cells treated for 1 h with $1.0\,\mu g\,ml^{-1}$ DOX alone was 55% and 34% respectively. Although these results may suggest resistance is due to diminished retention, when TFP was present during accumulation but not retention as carried out with cytotoxicity studies, DOX retention in B16-BL6/S and B16-BL6/DOX0.25 cells at 120 min was still only 54% and 40% respectively. It therefore appears that in the absence or presence of TFP, there is little correlation between cellular DOX levels and the associated cytotoxicity in progressively DOX-resistant B16-BL6 cells. Overall, comparison of our earlier observations with a >100-fold DOX-resistant model system (Ganapathi & Grabowski, 1983) and the present results on cytotoxicity (Figure 1 and Table I) and DOX accumulation (Figure 2) in progressively DOX-resistant cells suggest that the effects of TFP in modulating cytotoxicity but not accumulation is related to the degree of DOX-resistance.

The antitumour studies in mice though preliminary appear promising and demonstrate that the effects of TFP in modulating DOX cytotoxicity *in vitro* occur *in vivo* as well. The magnitude of response *in vivo* with DOX plus TFP at high levels of resistance with the B16-BL6/DOX0.25 cells were not as encouraging as the *in vitro* results, and this may be related to the fact that sufficiently high cellular levels of DOX are not achieved at the maximally tolerated dose and schedule of DOX used.

In summary, results from this study demonstrate that there is little correlation between cellular accumulation of DOX and magnitude of resistance in progressively DOX-resistant B16-BL6 cells. The calmodulin inhibitor TFP was appreciably more effective in enhancing cytotoxicity rather than accumulation of DOX in the progressively resistant sublines and the magnitude of TFP effects on DOX cytotoxicity was related to the level of resistance. The superior chemotherapeutic efficacy *in vivo* of DOX plus TFP compared to DOX alone against pulmonary metastases of DOX-resistant B16-BL6 cells may be relevant to the recent demonstration (Miller *et al.*, 1988) that TFP may be of therapeutic value clinically in modulating the cytotoxicity of DOX in tumours with acquired DOX-resistance.

Supported by PHS Grant Number RO1 CA35531 awarded by the National Cancer Institute, DHHS. The authors would like to thank Dr Gerald J. Beck, Department of Biostatistics and Epidemiology for helpful advice in the statistical analysis of the data. The excellent secretarial assistance of Nijole Mazelis and Robbie Martin and skilful preparation of the art work by Joseph Kanasz of the Art-Medical Illustrations and Photography Department is gratefully acknowledged.

### References

BACHUR, N.R., MOORE, A.L., BERNSTEIN, J.G. & LIU, A. (1970). Tissue distribution and disposition of daunomycin (NSC-82151) in mice: fluorometric and isotopic methods. *Cancer Chemother. Rep.*, **54**, 89.

CARTER, S.K. (1975). Adriamycin – a review. *J. Natl Cancer Inst.*, **55**, 1265.

DANO, K. (1976). Experimentally developed cellular resistance to daunomycin. *Acta Pathol. Microbiol. Scand. Suppl.*, **256**, 11.

GANAPATHI, R. & GRABOWSKI, D. (1983). Enhancement of sensitivity to Adriamycin in resistant P388 leukemia by the calmodulin inhibitor trifluoperazine. *Cancer Res.*, **43**, 3696.

GANAPATHI, R. & GRABOWSKI, D. (1988). Differential effect of the calmodulin inhibitor trifluoperazine in modulating cellular accumulation, retention, and cytotoxicity of doxorubicin in progressively doxorubicin resistant L1210 mouse leukemia cells. Lack of correlation between cellular doxorubicin levels and expression of resistance. *Biochem. Pharmacol.*, **37**, 185.

GANAPATHI, R., GRABOWSKI, D., TURINIC, R. & VALENZUELA, R. (1984*a*). Correlation between potency of calmodulin inhibitors and effects on cellular levels and cytotoxic activity of doxorubicin (Adriamycin) in resistant P388 mouse leukemia cells. *Eur. J. Cancer Clin. Oncol.*, **20**, 799.

GANAPATHI, R., GRABOWSKI, D., ROUSE, W. & RIEGLER, F. (1984*b*). Differential effect of the calmodulin inhibitor trifluoperazine on cellular accumulation, retention, and cytotoxicity of anthracyclines in doxorubicin (Adriamycin)-resistant P388 mouse leukemia cells. *Cancer Res.*, **44**, 5056.

GANAPATHI, R., GRABOWSKI, D., SCHMIDT, H., SESHADRI, R. & ISRAEL, M. (1985). Calmodulin inhibitor trifluoperazine selectively enhances cytotoxic effects of strong *vs.* weak DNA binding antitumour drugs in doxorubicin-resistant P388 mouse leukemia cells. *Biochem. Biophys. Res. Comm.*, **131**, 912.

GANAPATHI, R., YEN, A., GRABOWSKI, D., SCHMIDT, H., TURINIC, R. & VALENZUELA, R. (1986). Role of the calmodulin inhibitor trifluoperazine on the induction and expression of cell cycle traverse perturbations and cytotoxicity of daunorubicin and doxorubicin (Adriamycin) in doxorubicin-resistant P388 mouse leukemia cells. *Br. J. Cancer*, **53**, 561.

GANAPATHI, R., GRABOWSKI, D., SCHMIDT, H., BELL, D. & MELIA, M. (1987). Characterization *in vitro* and *in vivo* of progressively Adriamycin-resistant B16-BL6 mouse melanoma cells. *Cancer Res.*, **47**, 3464.

GERLACH, J.H., ENDICOTT, J.A., JURENKA, P.F. & 4 others (1986). Homology between P-glycoprotein and a bacterial haemolysin transport protein suggests a model for multidrug resistance. *Nature*, **324**, 485.

HARKER, W.G., BAUER, D., ETIZ, B.B., NEWMAN, R.A. & SIKIC, B.I. (1986). Verapamil-mediated sensitization of doxorubicin-selected pleiotropic resistance in human sarcoma cells: selectivity for drugs which produce DNA scission. *Cancer Res.*, **46**, 2369.

INABA, M., KOBAYASHI, H., SAKURAI, Y. & JOHNSON, R.K. (1979). Active efflux of daunorubicin and Adriamycin in sensitive and resistant sublines of P388 leukemia. *Cancer Res.*, **39**, 2200.

KESSEL, D. & WILBERDING, C. (1985). Anthracycline resistance in P388 murine leukemia and its circumvention by calcium antagonists. *Cancer Res.*, **45**, 1687.

KRISHAN, A., SAUERTEIG, A. & WELLHAM, L.L. (1985). Flow cytometric studies on modulation of cellular Adriamycin retention by phenothiazines. *Cancer Res.*, **45**, 1046.

MILLER, R.L., BUKOWSKI, R.M., BUDD, G.T. & 5 others (1988). Clinical modulation of doxorubicin resistance by the calmodulin-inhibition trifluoperazine: A Phase I/II trial. *J. Clin. Oncol.*, **6**, 880.

MYERS, C.E. (1982). Anthracyclines. In *Pharmacologic Principles of Cancer Treatment*, Chabner, B. (ed) p. 416. W.B. Saunders: Philadelphia.

NETER, J. & WASSERMAN, W. (1974). Analysis of covariance for completely randomized designs. In *Applied Linear Statistical Models*, p. 685, Richard D. Irwin: Homewood, Illinois.

RIORDAN, J.R. & LING, V. (1985). Genetic and biochemical characterization of multidrug resistance. *Pharmac. Ther.*, **28**, 51.

SIEGFRIED, J.M., TRITTON, T.R. & SARTORELLI, A.C. (1983). Comparison of anthracycline concentrations in S180 cell lines of varying sensitivity. *Eur. J. Cancer Clin. Oncol.*, **19**, 1133.

SKOVSGAARD, T. (1978). Mechanisms of resistance of daunorubicin in Ehrlich ascites tumor cells. *Cancer Res.*, **38**, 1785.

SLATER, L.M., MURRAY, S.L., WETZEL, M.W., WISDOM, R.M. & DuVALL, E.M. (1982). Verapamil restoration of daunorubicin responsiveness in daunorubicin-resistant Ehrlich ascites carcinoma. *J. Clin. Invest.*, **70**, 1131.

TSURUO, T., IIDA, H., TSUKAGOSHI, S. & SAKURAI, Y. (1982). Increased accumulation of vincristine and Adriamycin in drug-resistant P338 tumor cells following incubation with calcium antagonists and calmodulin inhibitors. *Cancer Res.*, **42**, 4730.

TSURUO, T., IIDA, H., TSUKAGOSHI, S. & SAKURAI, Y. (1983). Circumvention of vincristine and Adriamycin resistance *in vitro* and *in vivo* by calcium influx blockers. *Cancer Res.*, **43**, 2905.

WHEELER, C., RADER, R. & KESSEL, D. (1982). Membrane alterations associated with progressive adriamycin resistance. *Biochem. Pharmacol.*, **31**, 1691.